# Long-term breast cancer risk post childhood cancer radiotherapy: data-driven screening recommendations for survivors

**DOI:** 10.1093/bjr/tqaf131

**Published:** 2025-07-09

**Authors:** Charlotte Demoor-Goldschmidt, Mathilde Lefresne, Emmanuel Jouglar, Luc Ollivier, Stéphanie Bolle, Claire Alapetite, Gilles Truc, Laetitia Padovani, Anne Laprie, Valérie Bernier-Chastagner

**Affiliations:** Pediatric Oncology Department, University Hospital of Angers, Angers, 49000, France; Pediatric Oncology Department, University Hospital of Caen, Caen, 14000, France; Inserm Unit 1018, Gustave Roussy, Villejuif, 94800, France; Pediatric Oncology Department, University Hospital of Angers, Angers, 49000, France; Institut Curie, Radiation Oncology Departement, Paris & Proton Center, Orsay, 75005, France; Radiation Oncology Department, Institut de Cancérologie de l’Ouest, Saint Herblain, 44800, France; Radiation Oncology Department, Gustave Roussy, Villejuif, 94800, France; Institut Curie, Radiation Oncology Departement, Paris & Proton Center, Orsay, 75005, France; Department of Radiation Oncology, Georges François Leclerc Cancer Center, Dijon, 21000, France; INP, UMR7051, Marseille, 13005, France; Department of Radiotherapy, Assistance Publique Hôpitaux de Marseille, Marseille, 13385, France; Radiation Oncology Department, Institut Universitaire du Cancer de Toulouse (IUCT) Oncopole Claudius Regaud, Toulouse, 31059, France; Radiotherapy Department, Institut de Cancérologie de Lorraine, Vandoeuvre-les-Nancy, 54500, France

**Keywords:** childhood cancer survivor, breast screening, breast cancer, radiotherapy, guidelines

## Abstract

**Objectives:**

Modern radiotherapy techniques have significantly reduced radiation exposure to healthy tissues. This study analysed breast tissue radiation doses in a national pediatric radiotherapy database, explored the discrepancy between prescribed and actual doses and analysed shortcomings of current breast screening guidelines.

**Methods:**

We analysed PediaRT database data on radiotherapy dose distributions for 5079 French childhood cancer patients (2013–present), focusing on photon therapy plans with available breast dose data (*n* = 428). Biological equivalent dose (BED) was calculated, and statistical analyses (*t*-tests, chi-square, Mann–Whitney *U*, Fisher’s exact, and logistic regression) identified factors associated with breast dose exposure.

**Results:**

Among patients receiving supra-diaphragmatic radiotherapy, 99.3% had a prescribed dose >10 Gy, but 62.1% received a maximum dose greater than 10 Gy to at least one breast, and 25.5% received a mean dose greater than 10 Gy. As pediatric treatments often use fraction sizes <2 Gy, BED analysis revealed that 51.29% to 80.47% of patients prescribed >10 Gy received <10 Gy as minimal, maximal, or mean dose. These discrepancies suggest that current breast cancer screening guidelines, based on prescribed doses, may overestimate risk in survivors treated with modern techniques.

**Conclusions:**

Our findings support a shift toward dose-specific screening recommendations based on actual tissue exposure. Relying solely on prescribed doses and fields may lead to unnecessary screenings and increased burden for patients.

**Advances in knowledge:**

This study is the first to assess actual breast tissue radiation doses using a national multicenter pediatric radiotherapy database. It highlights how current breast cancer screening guidelines based on prescribed doses may overestimate risk in survivors treated with modern radiotherapy techniques.

## Background

Radiotherapy (RT) plays a crucial role in treating childhood cancers and significantly improves survival rates. However, exposure to radiation, particularly in the chest area, heightens the risk of secondary malignancies, such as breast cancer. This article reviews long-term breast cancer surveillance recommendations for childhood cancer survivors (CCS) who received radiotherapy, comparing international guidelines and referencing sources accessible via PubMed and Google Scholar.^1–3^

### Radiotherapy in childhood and breast cancer risk

Children treated for cancers such as Hodgkin lymphoma, Wilms tumor, or medulloblastoma may receive, depending on the stage and type of disease, radiotherapy to the chest or abdomen. Studies indicate that female survivors who underwent chest RT before age 30 face a significantly increased risk of breast cancer, with incidence rates comparable to those of BRCA mutation carriers.^4–9^ The risk is dose-dependent; higher doses correlate with greater risk.^10^ Distinguishing between the prescribed dose and the actual dose received by breast tissue is crucial, as it can significantly impact surveillance recommendations.

### International recommendations for breast screening cancer after radiotherapy

Table 1 summarizes the breast cancer screening recommendations for CCS across different countries, highlighting the starting age, frequency, and criteria based on radiation therapy exposure.

**Table 1.** Breast screening recommendations published on a national level.

Country/Region	Recommended screening	Start age	Frequency	Year of update	Radiation therapy (RT) indication	Additional notes	Link
United States	MRI and mammography	25 or 8 years post-RT	Annual	2018	≥20 Gy chest RT	Patient education emphasized	COG, V6.0, 2023
United Kingdom	MRI	25 or 8 years post-RT	Annual	2022	≥20 Gy chest RT	Focus on patient education and self-examination	CCLG, V4, 2022
Canada, Australia	MRI and mammography	25 or 8 years post-RT	Annual	2016	≥20 Gy chest RT	Aligned with COG	
	Clinical exam	<25 years + 8 years post RT	Annual				NCCN
NCCN	Clinical exam + mammogram with tomosynthesis + breast MRI with and without contrast ± whole breast ultrasound	≥25 years + 8 years post RT	/6-12 months Annual		RT with exposure to breast tissue between ages 10 and 30 years		

*COG*  http://www.survivorshipguidelines.org/pdf/2023/COG_LTFU_Guidelines_Only_v6.pdf.

*CCLG*  https://www.cclg.org.uk/.

*Australia*  https://childrenscancer.canceraustralia.gov.au/health-professionals-and-researchers/cancer-information-and-guidelines.

*NCCN*  https://www.nccn.org/professionals/physician_gls/pdf/breast-screening.pdf.

Abbreviations: MRI = magnetic resonance imaging; RT = radiotherapy.

Recently, the International Late Effects of Childhood Cancer Guideline Harmonization Group (IGHG) refined their recommendations:^10^

-For chest doses of 20 Gy or more: Annual mammograms and breast MRIs starting at age 25 or 8 years post-radiation, whichever comes later.
-For chest doses between 10 and 19 Gy: Consideration of individual risk factors to determine the appropriate starting age and frequency for surveillance.
-For chest doses below 10 Gy: Routine breast cancer surveillance as per general population guidelines, unless other risk factors are present.

While there is a consensus on the need for early and regular breast cancer screening among CCS who received chest RT, minor differences exist in the specifics of these guidelines across countries. The objective of this study is to analyse the dosimetric data from the PediaRT database, which includes all French pediatric radiotherapy dose distributions administered in the 20 authorized centers for childhood cancer, to determine the actual doses received by the breast tissue and evaluate the implications for breast cancer screening recommendations depending on the dose received and the prescribed dose, and analysed shortcomings of current breast screening guidelines.

## Method

We utilized the PediaRT database, which includes radiotherapy dose distributions from 20 French centers (excluding palliative treatments and total body irradiation) for childhood cancer patients (*n* = 5079) treated since 2013. We included all photon therapy treatment plans, even if multiple plans were delivered to the same patient, when doses received by the breasts or mammary buds (referred to as ‘breast’ in this article) were available (*n* = 428, of whom 354 were undergoing their first irradiation treatment). For patients receiving re-irradiation or a second treatment plan (*n* = 74), cumulative doses were not considered.

The analysis was conducted in several steps:

1. Comparison of included vs. nonincluded patients: We first compared patients with available breast dosimetry data (*n* = 428) to those without (*n* = 4651), to evaluate potential selection biases. Variables compared included age, sex, treatment field, prescribed dose, dose per fraction, number of radiotherapy treatments and center, which is a frequent bias in multicenter studies and which corresponds to inter-center differences in terms of data collection.
2. Analysis of breast contouring determinants: Among the full PediaRT cohort, we analyzed factors associated with whether the breast tissue was contoured. We compared patients with and without breast contouring using univariate analyses (*t*-tests, chi-square, Fisher’s exact, or Mann–Whitney *U* tests, as appropriate). We then used logistic regression to identify independent predictors of breast contouring, considering variables such as sex, cancer type, treatment field, treatment center, age at treatment, and prescribed dose.
3. Analysis of dose distributions in contoured cases: In the 428 patients with breast dosimetry data, we evaluated the actual radiation dose received by the breast tissue. We computed descriptive statistics for minimum, maximum, mean, and median dose (D50) received by at least one breast, stratified by treatment field and prescribed dose. To account for the biological effects of different fractionation schedules, the biological equivalent dose (BED) was calculated using the formula: BED = *N* × *d* × (1 + *d*/(*α*/*β*)), where *N* represents the number of fractions (treatment sessions), d is the dose per fraction, and *α*/*β* is the tissue-specific ratio, estimated to be between 3 and 4 Gy for breast tissue.^11^
4. Identification of factors associated with high breast exposure: We performed univariate and multivariate logistic regression analyses to identify predictors of a maximum dose >10 Gy to any breast. Analyses were conducted both in the full cohort with contoured breasts and within subgroups (e.g., supra-diaphragmatic and craniospinal fields). Variables included in the models were age, height, weight, prescribed dose, dose per fraction, and treatment field. Variables with *P* < .20 in univariate analysis were included in multivariable models, and *P* < .05 was considered statistically significant in final models.

These analyses were performed using SAS 9.4 software.

The study is part of the ‘Investissement d'Avenir’ HOPE‐EPI program (https://rnce.inserm.fr/, https://rnce.inserm.fr/wp-content/uploads/2024/04/CCOP-RNCE_NoteInformation_20240410-1.pdf), supported by the ANR (ANR-10-COHO-0009). This study was conducted in accordance with the ethical standards outlined in the Declaration of Helsinki, and the protocol was approved by the Institut de Cancérologie de Lorraine Ethics Committee. Informed consent was obtained from all patients or their legal guardians before participation. The study adhered to strict ethical guidelines to ensure patient confidentiality and data protection, with no discrimination based on gender, ethnicity, or socioeconomic status during patient recruitment and data collection.

## Results

Table 2 compares the data included in the analysis with those not included, showing a significant center effect (*P* < .0001). In multivariable analysis, factors influencing the contouring of the breasts include sex, irradiation field, and treatment center (*P* < .001), followed by the prescribed dose (*P* = .009) and dose per fraction (*P* = .23), whereas age at treatment (*P* = .79), year of treatement (*P* = .60) and the number of radiotherapy treatments (*P* = .73) were not significant. For example, regarding cranio-spinal irradiation, for centers that registered more than 30 dosimetries, breasts were contoured between 5.77% and 72.22% of the cases. In the supra-diaphragmatic subgroup, age was not significantly different between groups (*P* = .29), but the type of cancer was different, with increased contouring of the breast for Hodgkin lymphoma and nephroblastoma (*P* < .0001).

**Table 2.** Comparison of the data included in the analysis versus the others in the Pedia-RT cohort.

	Data on breast tissue available	Data on breast tissue not available	*P*
	*N* = 428	*N* = 4651	
Age at diagnosis (mean, [95%CI])	10.75 [10.24; 11.27]	9.48 [8.95; 10.17]	.25
Sex			<.001
Girl	336 (15.19)	1876 (96.79)	
Boy	92 (3.21)	2775 (84.81)	
Field			<.001
Cranial (*n* (%))	9 (0.50)	1793 (99.50)	
Cervical (*n* (%))	2 (0.78)	256 (99.22)	
Supra-diaphragmatic (*n* (%))	285 (30.82)	633 (69.18)	
Sub-diaphragmatic (*n* (%))	7 (0.61)	1136 (99.39)	
Cranio-spinal (*n* (%))	126 (16.94)	618 (83.06)	
Arm (*n* (%))	2 (2.99)	65 (97.01)	
Leg (*n* (%))	0 (0)	150 (100)	
Prescribed dose (mean, [95%CI])	34.98 [33.46; 36.50]	41.24 [40.76; 41.71]	<.001
Dose per fraction			<.001
≤1 Gy	37 (13.12)	245 (86.88)	
[1; 1.8[Gy	83 (11.34)	649 (88.86)	
[1.8; 2.2] Gy	290 (7.95)	3359 (92.05)	
]2.2; 3[Gy	2 (6.67)	28 (93.33)	
3 Gy	1 (1.02)	97 (98.98)	
>3 Gy	15 (5.21)	273 (94.79)	
Number of radiotherapy treatment			.01
1 (*n* (%))	354 (8.04)	4049 (91.96%)	
2 or more (*n* (%))	74 (10.95)	602 (89.05%)	

**Figure 1.**
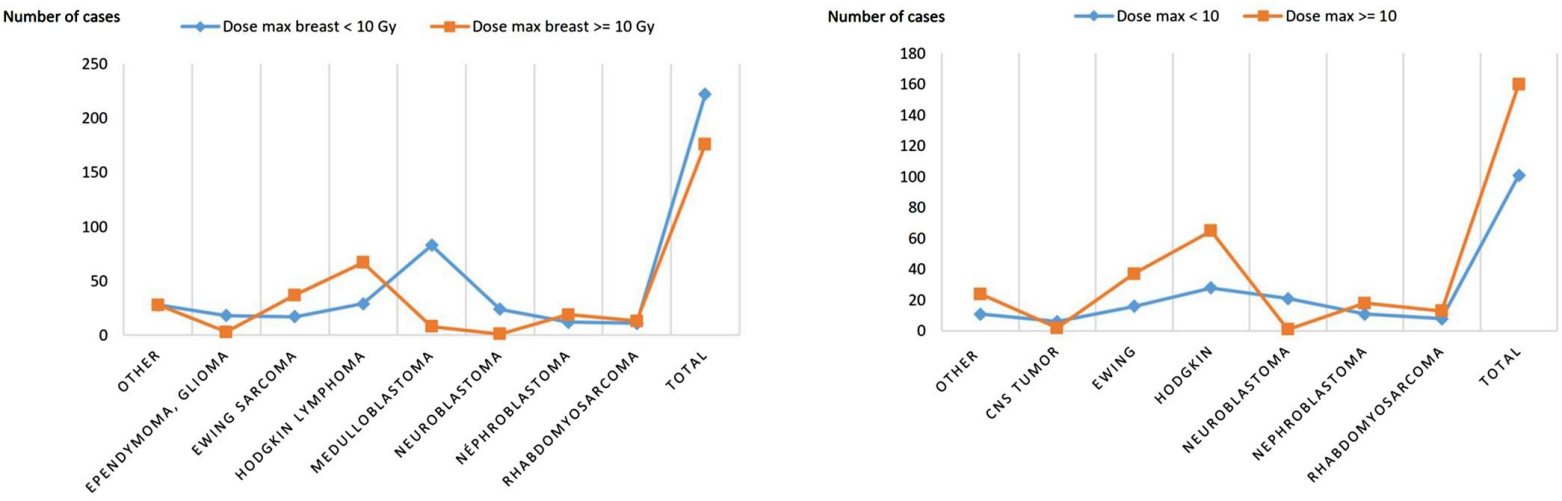
Repartition of the cancers regarding the maximal dose received on the breast tissue (A) in all the cohort (B) for patients treated with a supra-diaphragmatic field.

The radiation dose to breast tissue varies significantly depending on the cancer type, with patients treated for Hodgkin lymphoma and Ewing sarcoma, being the most commonly exposed to doses greater than 10 Gy, particularly in the supra-diaphragmatic field (Figure 1). Table 3 presents dosimetric data for breast tissue categorized by irradiation field and prescribed dose. For 99.3% (280/282) of treatments with at least one supra-diaphragmatic field, the prescribed dose was greater than 10 Gy. For these irradiations, the maximum dose to at least one breast exceeded 10 Gy in 62.1% of cases (60.2% when restricting the analysis to the first treatment), and it exceeded 5 Gy in 77.7% of cases (77.3% when restricting the analysis to the first treatment). The mean dose to at least one breast was greater than 10 Gy in only 25.5% of cases (21.9% when restricting the analysis to the first treatment). Among these 428 treatments, if considering the maximum dose received by the breast, screening would be recommended for 193 patients (being 45.1%, including 90.7% treated with a supra-diaphragmatic field); and 64.2% of these patients would not require screening if the dose received by 50% of the breast is considered. Additionally, 32 out of 69 cases (46.4%) with D50 > 10 Gy received a small dose per fraction (<1.8 Gy/fraction), which reduces the BED (Tables 3 and 4). On the other hand, only 15 out of 359 cases (4.2%) with D50 < 10 Gy received a high dose per fraction (>3 Gy). Nevertheless, in these cases, the breast tissue was not within the irradiated field, and applying BED calculations did not affect the number of patients whose D50 exceeded 10 Gy. When using the BED, since the dose per fraction was under 2 Gy per fraction for 85.98% of the cohort, between 51.29% and 80.47% of patients with a prescribed dose over 10 Gy in fact received less than 10 Gy (regardless of the characteristics used, be it minimum, maximum, or mean dose).

**Table 3.** Dosimetric analysis, PediaRT database.

Field	Prescribed dose			Mean age at treatment	Maximum dose received by the breast tissue *(on at least 1 breast)*		Minimum dose received by the breast tissue *(on at least 1 breast)*		Mean dose received by the breast tissue *(on at least 1 breast)*		Median dose (D50%) received by the breast tissue *(on at least 1 breast)*		Median BED (*α*/*β* = 4) (calculated on D50% *on at least 1 breast)*	Median BED (*α*/*β* = 3) (calculated on D50% *on at least 1 breast)*
	<10 Gy	[10-20[Gy	≥20 Gy		≥10 Gy	≥5 Gy	≥10 Gy	≥5 Gy	≥10 Gy	≥5 Gy	≥10 Gy	≥5 Gy	≥10 Gy	≥10 Gy
Cranial and cervical	0/11	0/11	11/11 (100%)	8.8 ± 4.7	3/11 (27.3%)	5/11 (45.5%)	0/11	0/11	0/11	0/11	0/11	0/11	0/11	0/11
Cranio-spinal	0/126	5/126 (4.0%)	121/126 (96.0%)	9.0 ± 4.3	13/126 (10.3%)	60/126 (47.6%)	8/126 (6.4%)	45/126 (36.6%)	1/126 (0.8%)	24/126 (19.1%)	1/126 (0.8%)	2/126 (1.6%)	109/126 (86.5%)	2/126 (1.6%)
Supra-diaphragmatic	2/282 (0.7%)	102/282 (36.2%)	178/282 (63.1%)	11.7 ± 5.6	175/282 (62.1%)	219/282 (77.7%)	157/282 (55.7%)	199/282 (70.6%)	72/282 (25.5%)	143/282 (50.7%)	68/282 (24.1%)	127/282 (45.0%)	81/282 (28.7%)	87/282 (30.9)
Sub-diaphragmatic	1/7 (14.3%)	2/7 (28.6%)	4/7 (57.1%)	12.0 ± 3.4	2/7 (28.6%)	3/7 (42.9%)	0/7	0/7	0/7	0/7	0/7	0/7	0/7	0/7
Arm	0	0	2 (100%)		0/2	0/2	0/2	0/2	0/2	0/2	0/2	0/2	1/2 (50%)	1/2 (50%)
Total	3/428 (0.7%)	109/428 (25.5%)	316/428 (73.8%)	10.8 ± 5.3	193/428 (45.1%)	287/428 (67.1%)	165/428 (38.6%)	287/428 (67.1%)	73/428 (17.1%)	167/428 (39.0%)	69/428 (16.1%)	150/428 (35.1%)	83/428 (19.4%)	89/428 (20.8%)

D50% (median dose): received dose by 50% of breast tissue volume.

**Table 4.** Biological equivalent dose on the breast tissue and prescribed dose, PediaRT database.

*α*/*β* = 4	BED max		BED min		BED mean	
Prescribed dose	<10 Gy	≥10 Gy	<10 Gy	≥10 Gy	<10 Gy	≥10 Gy
<10 Gy	2	1	3	0	3	0
[10-20[Gy	31 (28.44)	78 (71.56)	33 (30.28)	76 (69.72)	75 (68.81)	34 (31.19)
≥20 Gy	193 (61.08)	123 (38.92)	220 (69.62)	96 (30.38)	267 (84.49)	49 (15.51)
≥10 Gy	224 (52.71)	201 (47.29)	253 (59.53)	172 (40.47)	342 (80.47)	83 (19.53)

*α*/*β* = 3	BED max		BED min		BED mean	
Prescribed dose	<10 Gy	≥10 Gy	<10 Gy	≥10 Gy	<10 Gy	≥10 Gy
<10 Gy	0	3	3	0	3	0
[10-20[Gy	28 (25.69)	81 (74 .31)	33 (30.28)	76 (69.72)	71 (65.14)	38 (34.86)
≥20 Gy	190 (60 .13)	126 (39.87)	217 (66.67)	99 (31.33)	265 (83.66)	51 (16.14)
≥10 Gy	218 (51.29)	207 (48.71)	250 (58.82)	175 (41.18)	332 (78.12)	89 (20.94)

Multivariable analysis studying the risk of a maximum breast dose exceeding 10 Gy showed that patient’s age or height at treatment (*P* < .001) and the use of a supra-diaphragmatic field were significant risk factors (*P* = .03), but not the prescribed dose (Table 5). For patients treated with a supra-diaphragmatic field (*n* = 282), 37.9% received a maximum dose under 10 Gy to both breasts, the prescribed dose was not a significant factor in breast irradiation, unlike age and size (Table 5). Among patients treated with cranio-spinal radiotherapy, 47.6% received a maximum dose over 5 Gy to at least one breast, and 10.3% exceeded 10 Gy. Multivariable analysis in this subgroup yielded similar results. The type of radiotherapy technique (3D conformal versus any technique including intensity modulation) was not a significant factor in breast irradiation (*P* = .67) in univariate analysis. Breast dose data for radiotherapy involving an abdominal or pelvic field were available for only 7 patients, with 3 receiving more than 5 Gy (ages 9.1, 16.8 and 17.2 years) and 2 receiving 10 Gy (ages 16.8 and 17.2 years).

**Table 5.** Multivariate analysis evaluating the risk of breast tissue receiving more than 10 Gy.

	MODEL 1 (complete cohort)				MODEL 2 (complete cohort)				MODEL 3 (for craniospinal field)				MODEL 4 (for supra-diaphragmatic field)			
	Relative Risk	Confidence 95%		*P*	Relative Risk	Confidence 95%		*P*	Relative Risk	Confidence 95%		*P*	Relative Risk	Confidence 95%		*P*
Age	1.15	1.10	1.20	**<.001**					1.16	1.02	1.32	**.03**	1.149	1.092	1.209	**<.001**
Field (reference = cranial)				**<.001**				**<.001**								
Supra-diaphragmatic	5.82	1.18	28.74		5.135	1.02	25.67									
Sub-diaphragmatic	0.58	0.04	8.71		0.61	0.04	9.15									
Cranio-spinal	0.55	0.10	3.11		0.47	0.08	2.68									
Prescribed dose	1.00	0.98	1.01	**.007**	1.00	0.98	1.02	.90	0.99	0.95	1.04	.94	0.997	0.978	1.015	.75
Height of the patient					1.02	1.01	1.03	**<.001**								

Bold indicates significant results.

## Discussion

This study provides a comprehensive analysis of breast radiation exposure in a large cohort of pediatric cancer patients treated with radiotherapy. Using the PediaRT database, which includes radiotherapy dose distributions from 20 centers in France. Our results show that patient and treatment characteristics—particularly age, height, and the use of a supra-diaphragmatic field—significantly influence the likelihood of breast tissue receiving doses greater than 10 Gy. Notably, 40% of patients with a supra-diaphragmatic field and a prescribed dose above 10 Gy actually received less than 10 Gy to any part of either breast. This proportion increases to over 50% when considering the BED. Furthermore, 23% received less than 5 Gy, challenging the rationale for classifying these patients as high-risk based solely on prescribed dose. These findings suggest that in modern treatment settings, relying only on prescribed doses and field locations may lead to an overestimation of breast cancer risk. It is also important to note that current guidelines do not take into account the impact of fractionation. Reirradiation appeared to be associated with higher breast exposure, but this may reflect a selection bias: such treatments often involve smaller fields located near the breast, making breast dose data more readily available.

While the maximum dose received by any part of the breast is a useful indicator of whether a hotspot exists, its biological relevance remains uncertain when that dose is limited to a very small tissue volume. Unlike toxicity endpoints where a focal overdose may lead to functional impairment, the risk of radiation-induced carcinogenesis is more often associated with the cumulative and distributed exposure to the organ. Most published dose–response models for secondary breast cancer, such as those by Inskip et al and Veiga et al, rely on broader metrics like the mean dose to the breast, or the estimated dose at the tumor location, rather than point maximum values. Currently, there is no validated threshold of cancer risk based on small-volume (e.g., voxel-level) high-dose exposure. Therefore, we chose to present both the maximum dose and the D50 (dose received by 50% of the breast volume). While the maximum dose indicates whether a part of the breast was exposed to a high dose, the D50 provides information about the extent of the tissue affected–which may be more relevant when assessing overall carcinogenic risk. From a clinical perspective, using only the maximum dose to guide screening may result in over-screening—potentially causing psychological stress, unnecessary follow-up exams, and financial burden, without a clear benefit. A volume-based assessment offers a more balanced and patient-centered approach.

In our study, we encountered significant gaps in the availability of precise dosimetric data for breast tissue, particularly for patients treated with abdominal or pelvic fields but also for those treated with supra-diaphragmatic or cranio-spinal fields. The lack of consistent and comprehensive data collection on breast dose across different radiotherapy centers complicates the assessment of true radiation exposure. When breast tissue is not nearby the field, dosimetric data are often not meticulously recorded. This inconsistency can lead to an overestimation of the percentage of patients who have breast irradiation when considering only supra-diaphragmatic field, and an underestimation of the dose received by the breast, especially in younger patients with abdominal fields where the mammary buds might still receive substantial radiation due to their proximity to other treatment areas. In response to these challenges, the adoption of artificial intelligence (AI) in the field of radiation oncology presents a promising solution, particularly through automated contouring techniques. These advanced technologies are capable of recognizing and delineating anatomical structures consistently across different scans, thereby mitigating the risk of human error and variability among centers.^19^ By integrating AI into routine clinical practice, we can bridge the gap in data collection quality, ensuring that every patient’s dosimetric data is meticulously captured and analysed. This approach is crucial for younger patients, where mammary buds are at risk of receiving inadvertent radiation, highlighting the importance of precise, AI-enhanced contouring to guide more accurate screening and follow-up protocols.

In the past two decades, rapid technological advancements have revolutionized radiation therapy for pediatric cancer patients, enhancing the ability to spare normal tissues while preserving treatment efficacy. By decreasing the target zone, such as in Hodgkin lymphoma, and using more focused beams, the area of exposure has been limited, reducing irradiation to organs. This reduction was feasible due to innovations in photon therapy as with Intensity-modulated radiotherapy (IMRT) or volumetric-modulated arc therapy (VMAT), image-guided patient positioning, and motion management. Current radiotherapy techniques produce detailed dose-volume histograms (DVHs) for healthy organs, allowing precise assessment of radiation exposure and facilitating efforts to minimize damage to surrounding tissues. These DVHs enable clinicians to optimize treatment plans for better therapeutic outcomes and reduced side effects. However, these advancements also introduce new complexities in determining the most relevant dosimetric parameters (e.g., maximum dose, mean dose) that should guide screening guidelines. As treatment fields become more precise and exposure patterns evolve, it is critical that screening protocols adapt accordingly to reflect these changes.

When examining articles suggesting a high risk even with smaller doses (10-19 Gy), there is a mixture of prescribed doses and estimated doses on the breast (Table 6). With modern radiotherapy techniques, the absorbed dose to the breast is often lower than the prescribed dose due to advanced beam shaping and tissue sparing. It is important to emphasize that this discrepancy primarily applies to CT-based 3D planning. In contrast, during the 2D era–with AP/PA or lateral fields–large portions of the breast were often within the treatment fields, and thus, likely received doses closer to the prescribed values, especially when overlapping with the target volume. However, even in these cases, the actual absorbed dose could still vary due to patient anatomy and tissue depth. These studies reaffirm that breast cancer risk increases with radiation exposure—but also highlight that modern mediastinal techniques can now spare the breast more effectively. In our study, 99.3% of patients with a supra-diaphragmatic field had a prescribed dose above 10 Gy, yet 48.4% had no breast tissue exposed above that threshold, and only 25.4% had a mean dose >10 Gy. Although treatment field remains the main predictor of breast dose, this underscores that prescribed dose and field alone are no longer sufficient criteria to determine screening eligibility.

**Table 6.** Breast cancer risk regarding the dose of radiotherapy in different published articles.

Article	Type of dose	Precision	Main results
Moskowitz et al *J Clin Oncol*. 2014^12^	Prescribed dose	14 Gy : whole lung (WL)	Cumulative incidence of breast cancer in women who received WL irradiation similar to those who received mantle field irradiation and elevated compared mediastinal field irradiation WLI, TBI: significant SIR
Veiga et al *JAMA Pediatr*. 2019^9^	Estimated dose received by the breast tissue where the breast cancer developed		Monovariable analysis: OR for overall breast cancer increased linearly with increasing radiation dose to breast cancer location, and it was significantly increased even for breast doses lower than 5 Gy (OR, 1.7; 95% CI, 1.0-3.0) compared with 0 Gy.
Guibout et al *J Clin Oncol*. 2005^13^	Mean dose to the breast		16 cases/1814 patients—analysis regarding intervals of doses not significant
Demoor-Goldschmidt et al *Radiother Oncol*. 2017^14^	3 Gy estimated to the breast, at least some part of the breast		No analysis in term of dose and risk factors
Ehrhardt et al *J Clin Oncol*. 2019^15^	Prescribed dose	Chest irradiation	0-10 Gy: HR 1.2 95%CI : 0.3-5.0 10-20 Gy: HR 8.0 95%CI 1.1-56.3 ≥20 Gy: HR 10.0 95%CI 3.3-30.5
Inskip et al *J Clin Oncol*. 2009^16^	Estimated dose received by the breast tissue where the breast cancer developed		1.3-11.3 Gy, OR 1.9 95%CI 0.7-5.0 11.4-29.99 Gy, OR 7.1 95%CI 2.9-17 30.0-60.0 Gy, OR 10.8 95%CI 3.8-31
Lange et al *Cancer*. 2014^17^	Prescribed and estimated dose	Wilms tumor	Numbers of survivors with invasive BC divided by numbers at risk : 16/369 (CR40 = 14.8% [8.7–24.5]) for women who received chest RT, 10/894 (CR40 = 3.1% [1.3–7.41]) if only abdominal RT (estimated received dose > 3 Gy) and 2/1229 (CR40 = 0.3% [0.0–2.3]) no RT
Taylor et al *Int J Cancer*. 2008^18^		Wilms tumor	No dose risk factor analysis

For patients treated recently, screening should be recommended after having analysed the detailed dosimetric information.

1. When precised dosimetric data on breast tissue does not exist, we invite professionals to consider to do a breast screening when the prescribed dose is over 20 Gy, in accordance with all guidelines.
  Breast screening is also recommended for smaller prescribed dose (10 Gy) when the breasts are certainly in-field, such as in total body or whole lung irradiation.
2. When precise breast dose data are available, although a clear threshold remains to be defined, a dose >10 Gy to any part of the breast may warrant consideration.

The lack of breast dose data in patients treated for abdominal or pelvic tumors is concerning, especially as 349 of our patients were under 4-year old. Due to their small size, their mammary buds may have been unintentionally exposed, even when the chest was not the primary target. This highlights a critical gap in screening strategies and the need for greater clinician awareness and education.

Finally, ethical considerations around screening should not be overlooked. The psychological and financial burdens of screening must be considered.^1^^,^^2^ Regular screening can cause significant stress and anxiety for patients, especially when they are subjected to frequent medical examinations and the associated fear of potential findings. Adherence to these recommendations is not optimal.^20^^,^^21^ The costs of these screenings also place a substantial financial strain on healthcare systems and families.^1^ In light of these concerns, our findings demonstrate that modern radiotherapy techniques, which more effectively spare healthy tissues, have significantly reduced actual radiation exposure to the breast. This suggests that current breast cancer screening guidelines, as well as the socio-economic models used to evaluate their cost-effectiveness, may overestimate the risk for CCS. Previous models, such as those used in the studies by Wong et al^1^ and Yeh et al,^2^ rely on older radiation exposure data that do not reflect the advancements in radiotherapy techniques. These models, which inform guidelines and healthcare policies, may, therefore, no longer be applicable to patients treated with contemporary methods, particularly when screening protocols are based on prescribed radiation doses and treatment fields. This could potentially lead to unnecessary screenings and associated costs.

Given these developments, updating these models with contemporary dosimetric data is essential.^22^^,^^23^ As shown in our study, shifting screening recommendations toward actual dose received—rather than prescribed dose—may optimize care, reduce unnecessary exams, and improve the efficiency of follow-up. In conclusion, this study underscores the complex interplay between the prescribed radiation doses and the actual exposure of breast tissue in pediatric cancer patients. While current guidelines advocate for systematic long-term breast screening with strong recommendation to perform breast cancer surveillance for survivors treated with ≥20 Gy chest radiation and moderate recommendation to perform breast cancer surveillance for survivors treated with 10-19 Gy chest radiation, our findings suggest that a significant portion of patients treated within the last decade may not receive sufficient exposure to justify such aggressive screening protocols. This emphasizes the need to modernize screening practices in line with current radiotherapy techniques. The integration of AI-based contouring and robust databases offers the opportunity to refine breast dose assessment, moving beyond simple field-based criteria. These innovations support more individualized, evidence-based screening recommendations that reflect real exposure and minimize unnecessary interventions. Furthermore, the availability of advanced radiotherapy databases facilitates the detailed analysis and application of these data, supporting the implementation of refined screening guidelines that are truly reflective of the doses absorbed by the tissues. As radiotherapy technology continues to evolve, it is imperative to continuously reassess and update screening guidelines to minimize unnecessary screenings and optimize care for survivors. This ongoing evolution will ensure that our screening protocols are not only based on the most accurate dose assessments but are also tailored to the real risk profiles of individual patients.

## Data Availability

The datasets generated and analyzed during the current study are not publicly available due to patient confidentiality, but are available from the corresponding author on reasonable request. Any requests for data access must comply with applicable ethical and legal standards, including data protection regulations and institutional review board approval.

## Code availability

The analyses performed in this study, including comparisons of means, chi-square tests, and logistic regression, were conducted using SAS 9.4 software. No custom code or algorithms were created for this study. However, the SAS script used for the analyses can be made available upon reasonable request by contacting the corresponding author.
